# Supplementary material for: Seminoma and Embryonal Carcinoma Footprints Identified by Analysis of Integrated Genome-Wide Epigenetic and Expression Profiles of Germ Cell Cancer Cell Lines
Source: PLoS One. 2014 Jun 2;9(6):e98330. doi: 10.1371/journal.pone.0098330 (PMC4041891; doi:10.1371/journal.pone.0098330)
Supplement: File S1 — ZIP file containing DMRforPairs output for significant regions. Please start from the html files. (ZIP) [file pone.0098330.s008.zip › figures/12040.pdf]

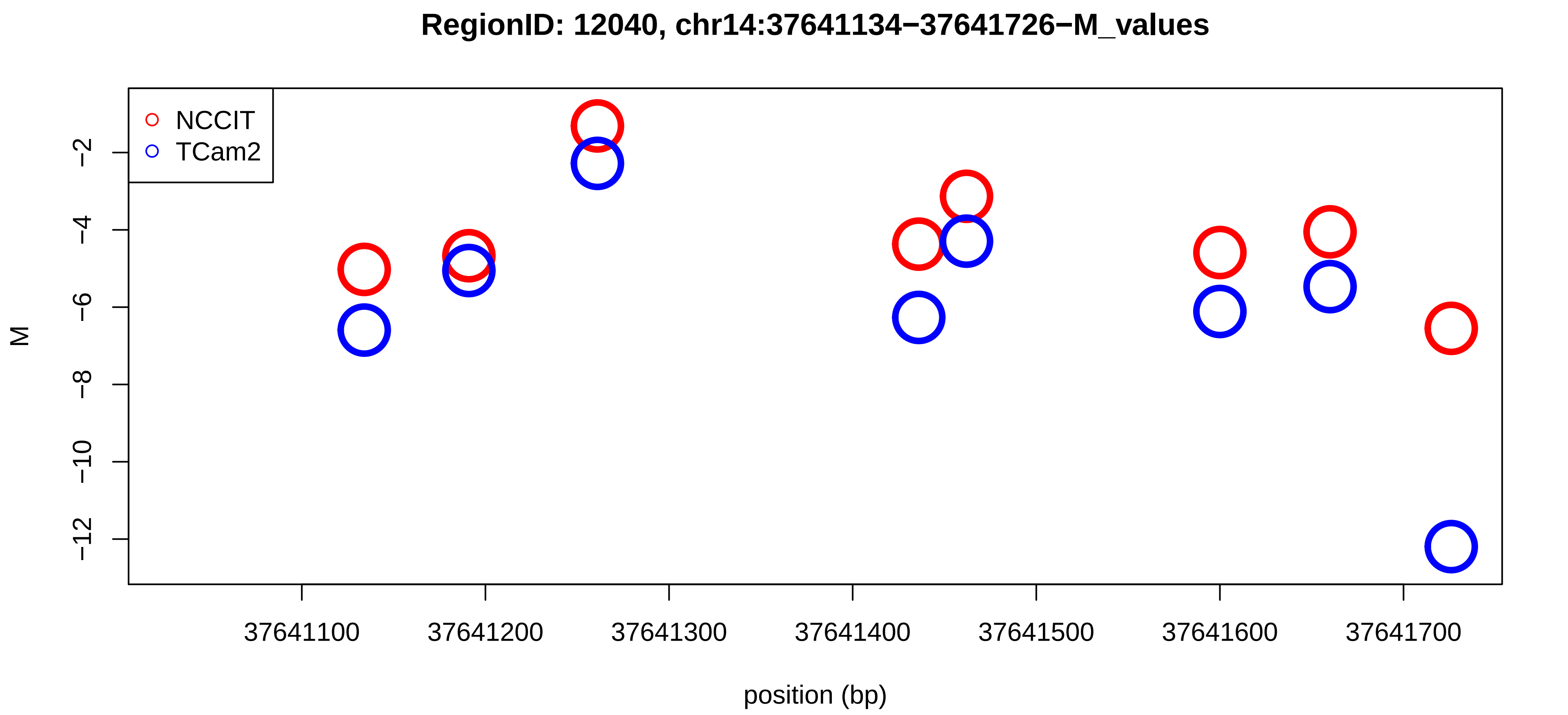

RegionID: 12040, chr14:37641134−37641726−M_values
NCCIT
TCam2
M
−2
−4
−6
−8
−10
−12
37641100
37641200
37641300
37641400
37641500
37641600
37641700
position (bp)

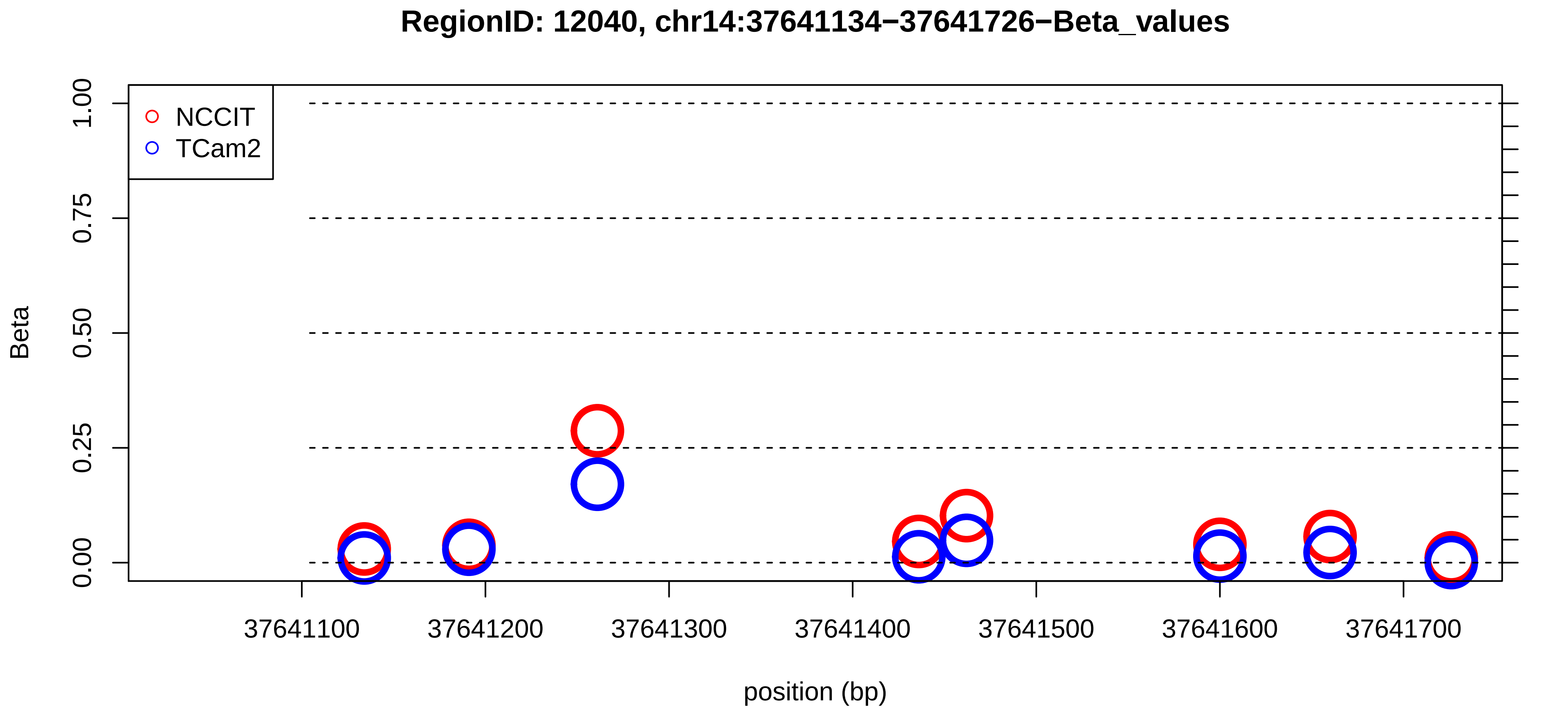

RegionID: 12040, chr14:37641134−37641726−Beta_values
NCCIT
TCam2
Beta
1.00
0.75
0.50
0.25
0.00
37641100
37641200
37641300
37641400
37641500
37641600
37641700
position (bp)
